# Regulatory effects of traditional Chinese medicine on the breast-cancer immune microenvironment

**DOI:** 10.3389/fimmu.2025.1706379

**Published:** 2026-01-15

**Authors:** Ting Liu, Jingying Shang, Jiameng Wang, Lutuo Han, Mingyue Yang, Ruonan Li, Baoyi Ni, Jiakang Jiang

**Affiliations:** 1 Heilongjiang University of Chinese Medicine, Harbin, China; 2 The Second Affiliated Hospital of Heilongjiang University of Chinese Medicine, Harbin, China; 3 The First Affiliated Hospital of Heilongjiang University of Chinese Medicine, Harbin, China

**Keywords:** breast cancer, immune evasion, immunotherapy, traditional Chinese medicine, tumor immune microenvironment

## Abstract

Breast cancer (BC) is the most common malignant tumor in women, driven by various factors. Its incidence has been rising annually and has become an urgent global public health challenge. An increasing body of evidence suggests that the immune microenvironment (IME) of breast cancer plays a crucial role in tumor initiation, progression, and metastasis. Through multi-target and multi-pathway regulatory effects, Traditional Chinese Medicine (TCM) demonstrates unique potential in reshaping the tumor immune microenvironment (TIME). This narrative review aims to systematically organize and summarize recent mechanistic advancements, elucidating how Traditional Chinese Medicine (primarily based on Chinese herbal medicine) regulates the function and polarization of key immune cells, controls immune checkpoints and cytokine networks, thereby inhibiting tumor immune escape, enhancing anti-tumor immunity, and exerting anti-BC effects. Despite the promising prospects, the application of TCM in BC immunotherapy still faces numerous challenges, including tumor heterogeneity, dosage complexity, and safety issues. Future research should focus on large-scale, multi-center clinical trials combining contemporary immunotherapy strategies, aiming to achieve better clinical outcomes in BC treatment and provide insights for immunotherapy of other cancer types.

## 1 Introduction

Breast cancer (BC) is the most frequently diagnosed malignancy in women and the second leading cause of cancer worldwide; according to the International Agency for Research on Cancer (IARC), more than 2.3 million new cases were recorded in 2022, representing 11.6% of all cancers (1). The global distribution of BC reflects a complex interplay of genetic, environmental and lifestyle factors. Incidence rates are generally higher in high-income countries than in low- and middle-income regions; however, absolute numbers of BC cases are rising in many developing nations owing to population growth and the adoption of Western lifestyles (2). Encouragingly, BC mortality is expected to decline as women gain broader access to advanced preventive measures, early diagnosis and medical intervention (3). Deciphering BC pathogenesis remains a central scientific priority, with recent investigations spanning tumor stemness, the intratumoral microbiome and circadian rhythms. Current evidence indicates that genetic susceptibility, hormonal exposure, nulliparity and unhealthy lifestyles cooperate to convert normal cells into malignant counterparts through stages of hyperplasia, premalignant lesions and carcinoma *in situ* (4).

The initiation, progression and metastasis of BC are intimately linked to its immune microenvironment (IME). The IME not only shapes tumor immune-evasion mechanisms but also actively modulates tumor progression (5). Tumour-infiltrating lymphocytes (TILs) play a pivotal role in BC immune surveillance, and their composition and function exert major prognostic influence (6). Principal immune infiltrates comprise T cells, dendritic cells (DCs), tumor-associated macrophages (TAMs), natural killer cells (NKs) and myeloid-derived suppressor cells (MDSCs) (7). The immune system can display dual roles by suppressing or promoting tumor growth (8). In BC, effector T cells and NK cells usually act anti-tumorally, constraining tumor expansion by recognizing and eliminating malignant cells (9, 10). However, immunosuppressive constituents of the tumor milieu—such as TAMs and MDSCs—secrete inhibitory mediators (e.g., IL-10 and TGF-β) that dampen anti-tumor immunity, thereby driving immune escape and metastasis (11, 12). TAMs are especially significant: via M2 polarization they enhance tumor growth, angiogenesis and immune evasion (13, 14). MDSCs facilitate invasion and dissemination by suppressing T-cell and NK-cell functionality (12). Remodeling of the IME is closely associated with the clinical prognosis of BC (15). The cellular composition and functional status of the IME decisively influence immune-escape mechanisms, metastatic capacity and therapeutic responsiveness (16). Consequently, re-establishing immune surveillance through IME modulation has become an important strategy in BC immunotherapy.

Prolonged clinical practice and abundant literature have shown that traditional Chinese medicine (TCM) enhances antitumor immunity, improves quality of life and prolongs median survival in cancer patients, underscoring its vast potential in tumor-immune modulation. TCM is a comprehensive therapeutic system that encompasses various treatment methods, including herbal medicine, acupuncture, moxibustion, and others. In this paper, we primarily discuss the role of herbal medicine in modulating the IME of BC. Although TCM treatments such as acupuncture show potential in certain aspects of immune regulation, they fall outside the scope of this discussion. TCM has a long history in treating BC; “Ru-yan” described in the classic text Furen Daquan Liangfang mirrors today’s clinical features of BC and was attributed to Liver–Spleen qi stagnation and qi-blood deficiency. In TCM theory, BC arises from qi stagnation, phlegm coagulation, blood stasis and toxic heat; therapy follows the principle of “supporting the upright and dispelling the evil,” emphasizing holistic, pattern-based regulation by tonifying qi, invigorating blood, resolving stasis, detoxifying, strengthening the Spleen and soothing the Liver to re-establish homeostasis (17). Contemporary studies further substantiate the therapeutic advantages of TCM in BC. For example, the classical formulas Yanghe Tang and Xihuang Wan have been shown to suppress tumor growth, modulate immunity and inhibit metastasis (18, 19). Moreover, Huangqi Sijunzi Tang confers pronounced benefits in BC patients with Spleen-Qi-deficiency-type cancer-related fatigue (CRF), alleviating clinical symptoms and markedly reducing physical, affective and cognitive fatigue scores (20). TCM likewise exhibits unique strengths in the prevention and treatment of precancerous lesions. Yanghe Huayan Decoction, one of the most widely used prescriptions for BC, has been proven capable of reversing breast carcinogenesis (21).

In summary, The TIME of BC is a complex network influenced by multiple factors, which enhances the adaptability of multi-target treatment approaches. Based on this, this review presents and argues a central thesis: the inherent multi-component, multi-target approach of TCM endows it with unique potential for systematically modulating this complex network.

## 2 Research objectives and methods

### 2.1 Research objectives

This review aims to systematically organize and describe recent advances in the mechanisms and research progress of TCM (primarily focusing on Chinese herbs) in modulating the tumor immune microenvironment (TIME) of BC. As a narrative review, its primary goal is to provide a comprehensive knowledge framework and an overview of the current state of the field, specifically including:

1. To summarize and categorize the potential targets, pathways, and key mechanisms through which TCM modulates the TIME of BC.
2. To integrate and present multi-level evidence from basic research to clinical translation.
3. To explore and forecast the potential of combining TCM with modern immunotherapies and the future research directions.

### 2.2 Literature search strategy

To comprehensively gather relevant literature, we conducted a systematic search across multiple databases:

Databases searched: PubMed, Web of Science, Embase.
Search period: April 2009 to November 2025.
Search keywords: (“breast cancer” OR “mammary carcinoma”) AND (“tumor immune microenvironment” OR “immunomodulation” OR “immune escape”) AND (“traditional Chinese medicine” OR “herbal medicine” OR “Chinese herbal formula”).

### 2.3 Inclusion and exclusion criteria

Inclusion Criteria:

1. The study topic involves the modulation of the BC IME by TCM (single herbs, formulations, or active ingredients).
2. Experimental studies (*in vivo* or *in vitro*), clinical studies (any design), and relevant mechanistic reviews.
3. The publication language is English.
4. The study provides clear data on mechanisms, outcomes, or clinical endpoints.

Exclusion Criteria:

1. Non-BC studies.
2. Studies not involving the IME or immune modulation mechanisms.
3. Conference abstracts, reviews, news articles, or non-research articles.
4. Incomplete data or inaccessible full text.

### 2.4 Literature selection and content synthesis process

This review adopts a narrative synthesis approach, with the following process:

1. Preliminary screening: Studies that do not meet the inclusion criteria are excluded based on titles and abstracts.
2. Full-text evaluation: Relevant literature is read in full and screened according to the inclusion/exclusion criteria.
3. Information extraction and theme construction: Key information (e.g., author, year, interventions, models/populations, main findings, mechanisms) is extracted from the final included studies. As a narrative review, this paper does not conduct formal quality scoring or bias risk assessment of the studies but aims to systematically describe and integrate the existing evidence through thematic synthesis (e.g., “immune cell regulation,” “signaling pathways,” “clinical combinatorial applications”), and identify knowledge gaps. Throughout the description, important limitations in study design (e.g., small sample size, non-randomized design) are pointed out.
4. Content organization: Based on the extracted information, the paper is organized in a logical sequence from basic mechanisms to clinical translation, constructing a coherent narrative flow to comprehensively reflect the current status and trends in the field.

## 3 The immune microenvironment and immune escape mechanisms in breast cancer

### 3.1 Basic composition of the immune microenvironment

Tumor cells, along with various immune cells, constitute the TIME, which includes tumor-associated immune cells, cytokines, and immune checkpoint molecules. This microenvironment has long been shown to be closely associated with tumor growth, recurrence, and metastasis (22, 23). Tumor-infiltrating lymphocytes are central to immune regulation: Cytotoxic (CD8^+^) T cells eliminate tumor cells by recognizing abnormal tumor antigens on cancer cells; CD4^+^ T cells differentiate into multiple subsets that coordinate various anti-tumor responses; and regulatory T cells (Tregs) exert anti-tumor immune effects through various mechanisms. For example, Tregs secrete growth factors such as TGF-β, which not only directly sustain tumor cell survival and proliferation but also promote tumor angiogenesis and broadly suppress anti-tumor immunity, thereby promoting tumor development (23–25). Within the tumor, infiltrating B cells can form tertiary lymphoid structures, present antigens to T cells, generate anti-tumor antibodies, and secrete cytokines to promote cytotoxic immunity. In contrast, regulatory B cells enhance immune suppression by secreting cytokines and transforming growth factor-β (TGF-β) (26). Innate lymphocytes provide an additional level of control: NK cells mediate MHC-independent lysis without damaging healthy allogeneic tissues, while γδ T cells possess characteristics of both innate and adaptive immunity. They can express or upregulate a large number of innate receptors under stimulation by IL-2 or IL-15, thereby conferring NK-like cytotoxic effects (27, 28). Among myeloid immune cells, M1 and M2 macrophages have opposing effects on tumors. M1 macrophages generate reactive oxygen species (ROS) and pro-inflammatory cytokines, playing a critical role in tumor cell killing, while M2 macrophages secrete IL-10 and TGF-β, promoting tumor progression (29, 30). Soluble mediators such as IFN-α, IFN-γ, IL-2, IL-12, IL-15, and granulocyte-macrophage colony-stimulating factor (GM-CSF) slow tumor growth by directly inhibiting cell proliferation and inducing cell death, or indirectly by enhancing anti-tumor immunity (31, 32). Immune checkpoint receptors such as PD-1, CTLA-4, LAG-3, and TIGIT, along with their ligands PD-L1 and CD80/86, constitute “brake” mechanisms that can be targeted by drugs (33). The synergistic interactions of components within the TIME influence tumor initiation, progression, and even resistance to treatment, thereby offering new avenues for cancer immunotherapy.

### 3.2 Immune escape mechanisms in breast cancer

BC evades immune surveillance through multiple mechanisms, including the establishment of an immunosuppressive tumor microenvironment, activation of immune checkpoints, and suppression of tumor antigen presentation (34). Specifically, BC creates a highly immunosuppressive tumor microenvironment (TME) to evade host immune surveillance. Hypoxia in the TME upregulates PD-L1 expression via HIF-1α, which suppresses the proliferation of cytotoxic T cells and promotes immune escape (35, 36). Tumor-associated macrophages (TAMs) recruit cancer-associated fibroblasts (CAFs) into the TME, forming an immunosuppressive environment that restricts intratumoral CD8^+^ T cell infiltration and increases resistance to immunotherapy (37–39). Immune checkpoint activation plays a central role in this escape process. The programmed cell death protein 1 (PD-1) pathway—comprising PD-1 and its ligand PD-L1—facilitates immune escape of BC cells by inhibiting T cell activation through receptor–ligand interaction (40). Clinical studies have shown that combining PD-1/PD-L1 inhibitors with chemotherapy significantly improves survival in PD-L1-positive patients with locally advanced or metastatic triple-negative BC (TNBC) (41). Immunosuppressive cell populations also play critical roles in BC immune escape. T cells progressively convert into suppressive regulatory T cells (Tregs), which inhibit effector T cell activity and accelerate BC development and progression (42). Myeloid-derived suppressor cells (MDSCs) impair the functions of T cells, dendritic cells (DCs), and NK cells by generating ROS, depleting arginine via arginase, and releasing immunosuppressive cytokines. High MDSC levels are therefore strongly associated with poor prognosis in metastatic BC (43). In addition, indoleamine 2,3-dioxygenase 1 (IDO1) promotes tumor immune tolerance and progression by converting tryptophan into kynurenine and increasing levels of immunosuppressive cells such as T cells and MDSCs (44). In summary, the immune escape mechanisms of BC are highly complex and heterogeneous, necessitating ongoing research and refinement of conceptual frameworks.

### 3.3 Heterogeneity of the immune microenvironment in different breast cancer subtypes

BC is a malignant tumor with high molecular heterogeneity, and its subtypes are clinically classified based on the expression of hormone receptors (ER), progesterone receptors (PR), and human epidermal growth factor receptor 2 (HER2). Different subtypes also exhibit significant differences in their TIME. TNBC typically has the most active immune microenvironment, often accompanied by a large accumulation of TILs. However, its genomic instability and higher somatic mutation burden make it more prone to immune escape and distant metastasis (45). In the TIME of this subtype, CD8^+^ T cells and regulatory Tregs play key roles (46). HER2-positive BC (HER2^+^) is one of the subtypes with the highest degree of immune cell infiltration. Its immune microenvironment undergoes significant dynamic changes under the combined influence of factors such as TILs, microsatellite instability, and tertiary lymphoid structures, particularly during HER2-targeted therapy, where such changes may lead to the development of resistance (47, 48). In contrast, the immune microenvironment of ER-positive BC (ER^+^) is typically in a suppressed state, with low levels of TILs. It may inhibit T cell function by expressing immune checkpoint molecules such as PD-L1, thereby weakening the anti-tumor immune response (49). In recent years, antibody-drug conjugates have emerged as a novel strategy for treating ER^+^ BC. By reducing immunogenic responses, they offer a new solution to overcome HER2 heterogeneity and resistance issues (50). Overall, the heterogeneity of the IME among different BC subtypes significantly affects the response to immunotherapy and clinical management of the disease. In the future, treatment strategies should be further optimized based on the immune characteristics of each subtype, promoting the development of personalized precision therapies.

## 4 Systematic modulation of the immune microenvironment of breast cancer by traditional Chinese medicine

**Figure 1:**
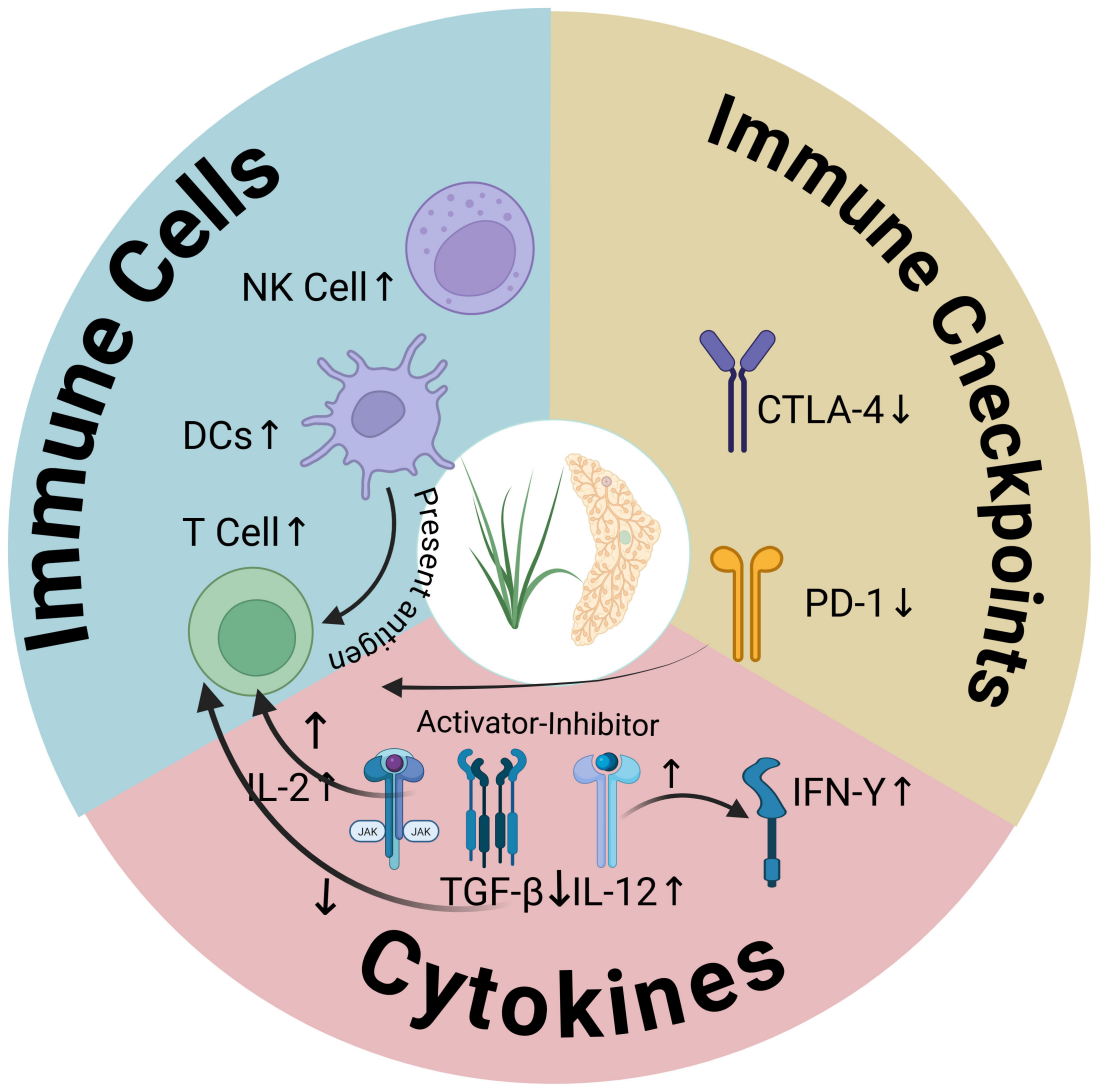
The regulatory effect of TCM on the main components of the TIME in BC. ↑Indicates promote; ↓indicates inhibit. Created in BioRender (https://BioRender.com).

In recent years, TCM has emerged as a powerful tool for modulating the TIME, primarily by enhancing immune cell activity and inhibiting immune suppressive factors to strengthen anti-tumor immunity (51). This is primarily due to the unique advantages of TCM, which involves multi-target and multi-pathway mechanisms, and its ability to enhance systemic anti-tumor capacity through multifaceted immune modulation, thereby demonstrating potential as an adjunctive therapy for conventional BC treatment (**Figure 1**, **Table 1**).

**Table 1:** Systematic regulation of breast cancer (BC) immune microenvironment by traditional chinese medicine (TCM).

Drug/Ingredient /Formulation	Dosage of TCM	Duration of treatment	Target/Cell/Pathway	Effect and related impact	Type of study	Reference
Yin nourishing and Qi-reinforcing Decoction (YFJP)	32.7 (high), 16.4 (medium), and 8.2 (low) g/kg	14 days	CD3^+^, CD8^+^ T cells; inhibitory receptors PD-1, TIGIT, TIM-3	Promotes T cell proliferation, downregulates inhibitory receptors, restores T cell cytotoxicity, inhibits tumor progression	*In vivo* (BALB/c mice)	(52)
Astragalus Polysaccharide	3 mg/kg	Once every 4 days until the study endpoint.	CD8^+^ T cells	Increases CD8^+^ T cell infiltration in tumor tissues, activates anti-tumor immune response	*In vivo* (BALB/c mice)	(53)
Saikosaponin A	35 mg/kg	56 days	IL-12/STAT4 pathway; CD4^+^, CD8^+^ T cells; IFN-γ, IL-12, IL-4, and IL-10	Increases T cell infiltration in tumors, activates IL-12/STAT4 pathway, upregulates IFN-γ and IL-12, downregulates IL-4 and IL-10, drives Th1 immune response, enhances anti-BC immunity	*In vivo*(SD rat)	(17, 54)
Ginseng Saponin Rh2 (GRh2)	/	/	ERp5; NKG2D-MICA signaling axis	Enhances NK cell activity, slows BC development and metastasis	*In vitro* (4T1)	(55)
GanoOil	6 mg/day	21 days	NK cells, CD8^+^ T cells; IL-6	Enhances NK cell and CD8^+^ T cell activity, promotes IL-6 secretion, inhibits BC lung metastasis	*In vitro*(4T1)	(56)
Xianlinglianxia Formula (XLLXF)	5.26 mg/kg	28 days	AKT1/STAT5 signaling axis; CISH protein; NK cells	Increases NK cell proportion and quantity, improves patient survival; activates AKT1/STAT5 signaling axis, downregulates CISH, enhances NK cell anti-tumor activity	*In vitro* and *in vivo* (BALB/c mice; SK-BR-3 and JIMT-1)	(57, 58)
Sophora Japonica Polysaccharide	50 mg/day	21 days	PI3K/Akt1 pathway; p38 MAPK; dendritic cells	Promotes dendritic cell maturation and function, inhibits p38 MAPK expression, enhances dendritic cell-mediated T cell activation, delays triple-negative BC progression	*In vitro* and *in vivo* (BALB/c(H-2^d^); DC2.4)	(60, 61)
Plantago Polysaccharide (PLP)	10 mg/kg	/	Dendritic cells; IL-12 p70, TNF-α	Promotes dendritic cell maturation, accelerates IL-12 p70 and TNF-α production, indirectly stimulates lymphocyte proliferation, triggers anti-tumor response	*In vivo* (C57BL/6 mice)	(62)
Matrine	/	/	TLR7/8 signaling pathway; dendritic cells; IL-12, TNF-α	Promotes IL-12, TNF-α release, promotes Th1 immune response, enhances NK cell and CTL cytotoxicity	*In vitro* (LLC)	(68)
Glycyrrhizin Polysaccharide-1 (GPS-1)	600 mg/kg	/	IFN-γ; CD3^+^, CD4^+^ T cells; IgG, IgA	Increases serum IFN-γ, CD3^+^ and CD4^+^ T cell proportion, enhances IgG and IgA concentrations, enhances cell-mediated and humoral immunity	*In vivo* (chickens)	(70)
Astragalus Polysaccharide	10 mg/kg	14 days	IFN-γ; Th1/Th2 balance	Enhances IFN-γ and related mediators, restores Th1/Th2 immune balance, enhances anti-BC defense	*In vivo*(BALB/c mice)	(71)
Dandelion Extract	/	/	TGF-β; Treg differentiation	Reduces TGF-β secretion, inhibits Treg differentiation, exerts anti-BC immune suppression	*In vivo* (BALB/c mice)	(73)
Berberine	4 mg/kg	14 days	PD-1, PD-L1; IFN-γ, Granzyme B	Downregulates PD-1 and PD-L1, promotes IFN-γ and Granzyme B production, enhances T cell activation and proliferation, activates TIME	*In vivo* (C57BL/6 mice)	(78)
Isoflavones combined with PD-1/PD-L1 antibodies	/	/	PD-1/PD-L1; CD4^+^, CD8^+^ T cells; IFN-γ	Enhances sensitivity to antibody therapy, promotes T cell infiltration, increases IFN-γ expression, reshapes BC TIME	*In vivo* (4T1 mice)	(80)
Cordycepin combined with anti-CTLA-4 drug	/	/	CTLA-4; T cells	Lowers CTLA-4 levels, accelerates T cell-mediated tumor clearance	*In vivo*	(82)

### 4.1 Modulation of immune cells in the breast cancer immune microenvironment by traditional Chinese medicine

The immune modulation potential of TCM has increasingly gained attention, especially the irreplaceable roles of key immune cells such as T cells, NK cells, and dendritic cells in the BC IME. Studies have shown that TCM can promote the activation and differentiation of CD4^+^ and CD8^+^ T cells, which are crucial for effective anti-tumor immunity. For example, the formula Yang Yin Fu Zheng Tang (YFJP), composed of Codonopsis pilosula, Adenophora triphylla, and Astragalus membranaceus, promotes the proliferation of CD3^+^ and CD8^+^ T cells in the peripheral blood and tumor tissues of C57BL/6 mice, and restores T cell cytotoxicity by downregulating inhibitory receptors such as PD-1, TIGIT, and TIM-3, thereby inhibiting tumor progression (52). Notably, Astragalus contains a variety of active components, such as polysaccharides, saponins, and flavonoids, all of which have been shown to possess immune-regulatory and anti-tumor effects. In the TNBC mouse model, Astragalus polysaccharides increase the infiltration of CD8+ T cells into the tumor tissue, triggering an anti-tumor immune response (53). Chaihu saponin A, an active component derived from the root of Bupleurum, has been shown to increase the infiltration of CD4^+^ and CD8^+^ T cells into BC tumors, thereby enhancing anti-BC immunity (17). Experiments in SD rats have verified that this effect is mediated through the activation of the IL-12/STAT4 pathway, which upregulates IFN-γ and IL-12, while reducing IL-4 and IL-10, thereby shifting the Th1/Th2 balance towards a Th1-type immune response (54). TCM also enhances NK cell cytotoxicity and prevents tumor immune escape. For example, ginsenoside Rh2 (GRh2) directly binds with ERp5 in MDA-MB-231x cells and modulates the NKG2D-MICA signaling axis to enhance NK cell activity, slowing the development and metastasis of BC (55). In the 4T1 mouse BC cell experiment, GanoOil significantly enhances NK cell and CD8+ T cell activity and promotes IL-6 secretion, playing an indispensable role in the treatment of BC lung metastasis (56). Moreover, a cohort study involving 844 HER2-positive BC patients showed that combined intervention with Xianling Lianxia Formula (XLLXF) on top of conventional treatment significantly increased the proportion and absolute count of NK cells in peripheral blood. The five-year survival rate in the combination therapy group (96.3%) was also improved compared to the trastuzumab-only group (88.7%). Further cellular and animal experiments revealed that XLLXF may alleviate the tumor microenvironment’s suppression of immune function by activating the AKT1/STAT5 signaling axis and downregulating the expression of CISH protein, ultimately enhancing NK cell anti-tumor activity (57). Network pharmacology analysis suggested that the tumor-immunomodulatory effects of XLLXF may be related to its active ingredients such as curcumin and icariin-II, providing a potential molecular theoretical basis for understanding how this formulation enhances NK cell function and improves patient survival prognosis (58). Dendritic cells are key to initiating adaptive immune responses, as they present antigens to T cells and trigger immune responses (59). *In vitro* experiments demonstrated that the main active ingredient of pagoda tree, pagoda tree polysaccharides, promotes dendritic cell maturation and function through the PI3K/Akt1 pathway, and inhibits p38 mitogen-activated protein kinase (MAPK) expression, thereby enhancing dendritic cell-mediated T cell activation and boosting anti-BC immunity (60). Further *in vivo* experiments confirmed that pagoda tree polysaccharides stimulate anti-tumor immune responses by increasing the proportion of mature dendritic cells, thereby prolonging the tumor-free survival time of xenografted mice and delaying TNBC progression (61). Advanced studies have shown that components extracted from Plantago polysaccharides (PLP) promote the maturation of dendritic cells in 4T1 mice, accelerate the production of IL-12 p70 and TNF-α, and indirectly stimulate lymphocyte proliferation, thereby triggering anti-tumor responses (62). Overall, TCM demonstrates significant immune modulation potential in the BC IME, exerting its unique anti-tumor effects by enhancing T cell activation, NK cell cytotoxicity, and dendritic cell function.

### 4.2 Modulation of immune mediators in the breast cancer immune microenvironment by traditional Chinese medicine

TCM can also enhance anti-tumor immune responses by modulating key immune mediators, such as cytokines and immune checkpoint molecules, offering promising prospects for BC immunotherapy.

#### 4.2.1 Modulation of cytokines

Cytokines such as IL-2, IL-12, IFN-γ, and TGF-β play key roles in tumor immune responses (63). However, the production of these cytokines is often suppressed, leading to immune escape (64). Both single compounds and multi-herb formulations in TCM can regulate the secretion and activity of these cytokines, thereby enhancing anti-tumor immunity (65). TCM has been reported to increase IL-2 secretion, promoting T cell proliferation and activation (66). For example, a study on BALB/c female nude mice confirmed that 4 weeks of continuous XLLXF administration significantly increased the levels of key cytokines such as IL-2 and IL-15 in peripheral serum, thereby enhancing T cell-mediated anti-tumor immune responses and ultimately inhibiting *in vivo* BC cell growth (58). IL-12 is critical for Th1 differentiation and the induction of IFN-γ (67). *In vitro* studies have demonstrated that Matrine promotes the release of cytokines such as IL-12 and TNF-α by regulating the TLR7/8 signaling pathway in dendritic cells, thereby promoting the Th1 immune response (68). This regulatory effect enhances the cytotoxic function of NK cells and CTLs, promoting tumor clearance (69). IFN-γ also plays an important role in anti-tumor immunity. *In vivo* experiments have confirmed that Glycyrrhizin polysaccharide-1 (GPS-1) significantly enhances anti-tumor immunity in chicks: on one hand, it increases cellular immunity, as evidenced by increased IFN-γ secretion and higher proportions of CD3^+^ and CD4^+^ T cells in serum; on the other hand, it enhances humoral immunity, leading to significant increases in IgG and IgA concentrations (70). Additionally, a key active ingredient in the herbal medicine Astragalus, Astragalus polysaccharides, can restore the Th1/Th2 immune balance in BALB/c mice carrying breast tumors by enhancing IFN-γ and related mediators, which is a crucial component of anti-breast cancer defense (71). TGF-β, as an immunosuppressive and anti-inflammatory cytokine, plays an irreplaceable role in tumor progression (72). In early tumor immune responses, TGF-β enhances anti-tumor immunity by regulating the differentiation and function of Tregs. Studies have shown that in the 4T1 mouse model, a key active ingredient extracted from Isodon rubescens, Isodon rubescens alkaloid, can inhibit Treg differentiation by reducing TGF-β secretion, thereby exerting an anti-breast cancer immunosuppressive effect (73). However, as the tumor progresses to advanced stages, TGF-β exhibits a clear dual role: it promotes cancer progression through various mechanisms such as inducing epithelial-mesenchymal transition (EMT), regulating the expression of corresponding transcription factors, and promoting immune escape (74). This suggests that TGF-β has dual roles in different immune environments, and the specific mechanisms require further in-depth research to uncover its potential clinical applications and challenges in tumor therapy.

#### 4.2.2 Regulation of immune checkpoint molecules

Immune checkpoints such as programmed cell death protein 1 (PD-1) and cytotoxic T lymphocyte-associated antigen 4 (CTLA-4) are key regulators of immune tolerance, and tumors exploit these molecules to evade immune surveillance (75). In recent years, TCM has been shown to modulate these checkpoints, thereby enhancing immune surveillance against BC (76). The PD-1/PD-L1 axis, comprising PD-1 and its ligand PD-L1, facilitates tumor immune escape by inhibiting T cell activation (77). *In vivo* studies in mice have shown that berberine downregulates PD-1 on T cells and PD-L1 on tumor cells, promotes the production of IFN-γ and granzyme B, thereby enhancing T cell activation and proliferation, and ultimately reactivating the TIME (78). This regulatory effect can enhance the efficacy of immune checkpoint inhibitors (ICIs) and restore immune surveillance in BC (79). Combination therapy using neoiso-flavones and PD-1/PD-L1 antibodies not only enhances the sensitivity of 4T1 mice to these antibodies but also promotes CD4^+^ and CD8^+^ T cell infiltration and boosts the expression of immune effector molecules such as IFN-γ, thus remodeling the BC TIME (80). Similarly, CTLA-4–targeted immunotherapy has been widely applied in BC treatment (81). Studies have shown that certain TCM components can reduce CTLA-4 expression, thereby promoting T cell activation and enhancing anti-tumor immunity (65). In the MC38 tumor model, co-administration of cordycepin and anti-CTLA-4 agents further reduced CTLA-4 levels and accelerated T cell–mediated tumor clearance (82). By finely modulating these immune checkpoints, TCM can overcome tumor-induced immunosuppression and enhance the efficacy of immunotherapy (83). Through the modulation of immune mediators, TCM exerts anti-tumor activity and offers novel and promising avenues for BC immunotherapy.

#### 4.2.3 Unique advantages of traditional Chinese medicine

Compared with conventional biologics (e.g., abatacept or monoclonal antibodies) and JAK inhibitors (e.g., baricitinib or tofacitinib), TCM presents several notable advantages. These agents typically target a single immune pathway or checkpoint, which may lead to acquired resistance or immune escape over time (84, 85). Furthermore, long-term use may induce immune-related adverse events such as fever or rash, and some patients exhibit limited clinical benefit (86). In contrast, TCM operates via multi-component, multi-target, and multi-pathway mechanisms, enabling the coordinated modulation of various immune cells and mediators within the IME, ultimately reshaping the TIME (87). Moreover, under the TCM principle of “syndrome differentiation and individualized treatment,” prescriptions are tailored to each patient. This personalized approach tends to result in fewer side effects and allows for synergistic application alongside other immunotherapies—such as immune checkpoint inhibitors or adoptive cell therapy—thereby enhancing efficacy while reducing treatment-related toxicity (88). In conclusion, TCM represents a promising adjuvant approach for BC immunotherapy, offering patients additional treatment options. When combined with modern immunotherapeutic strategies, it often demonstrates superior outcomes compared to conventional Western monotherapies.

## 5 Mechanistic insights into TCM-mediated modulation of the breast cancer immune microenvironment

**Figure 2:**
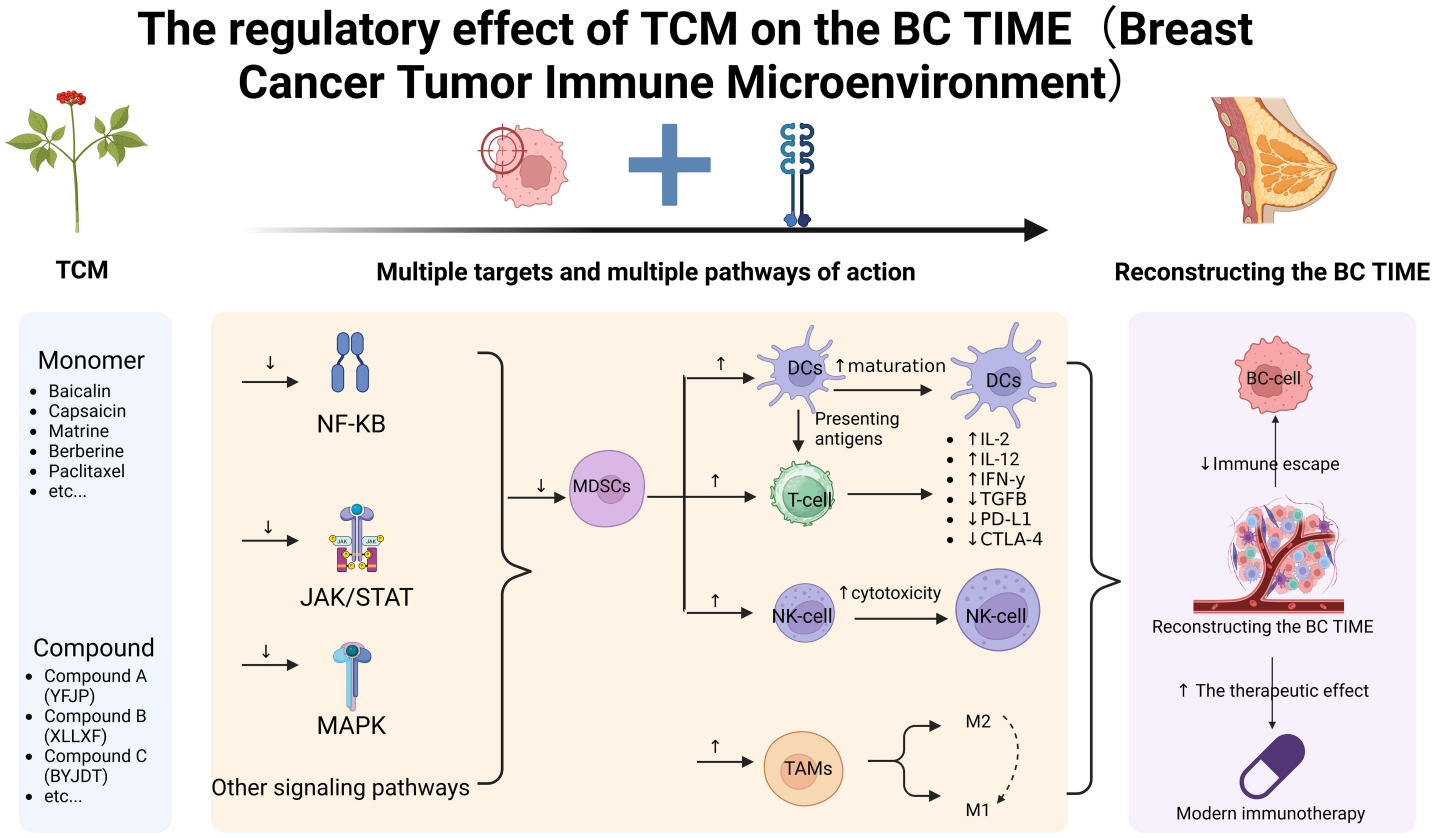
Mechanistic studies of TCM in modulating the BC TIME. ↑Indicates promote; ↓indicates inhibit. Created in BioRender (https://BioRender.com).

TCM exerts anti-tumor activity by remodeling the local IME, modulating systemic immune responses, and suppressing tumor cell proliferation, thereby offering a novel therapeutic avenue for patients with BC (51, 89). However, unlike single-target agents, the precise mechanisms of TCM remain incompletely elucidated, largely due to its multi-component nature and involvement in complex signaling networks (90, 91). This section provides a detailed analysis of these mechanisms, aiming to elucidate the scientific basis of TCM’s multi-target characteristics (**Figure 2**, **Table 2**).

**Table 2:** Mechanistic studies of traditional Chinese medicine (TCM) in modulating the breast cancer (BC) immune microenvironment.

TCM compound	Dosage of TCM	Duration of treatment	Target/Signaling pathway	Immunoregulation mechanism	Type of study	Reference
Quercetin	15.3 μg/ml	48h	IL-6/JAK2/STAT3 JAK/STAT, p53/Bax-Bcl-2	Inhibits IL-6/JAK2/STAT3 pathway, reduces Treg cell numbers, regulates TIME; inhibits JAK/STAT pathway, restores immune surveillance, enhances T cell recognition and clearance of cancer cells; upregulates p53 and pro-apoptotic protein Bax, downregulates anti-apoptotic protein Bcl-2 regulated by STAT3, induces BC cell apoptosis.	*In vitro* and *in vivo* (4T1、E0771、MCF-7; 4T1 mice)	(95, 114, 115, 117)
Resveratrol	/	21days	PD-1, macrophage polarization markers	Directly inhibits ER-negative BC cell proliferation; reduces PD-1 expression in T cells, promotes macrophage polarization to M1 phenotype, inhibits lung metastasis	*In vitro* and *in vivo*(BALB/c mice;4T1)	(96)
Hydroxymatrine + Astragaloside IV (liposome)	1.5 mg/kg	60 days	PD-1, CD8^+^ T cell functional markers (IFN-γ, Granzyme B)	Significantly enhances IFN-γ and Granzyme B expression in CD8^+^ TILs when combined with PD-1 inhibitors, enhances systemic anti-tumor immunity	*In vitro* and *in vivo* (BALB/c mice;4T1-luc)	(97)
Isoflavone	0–100 μmol/L	72h	IRF5/SLC7A5/IDO1	Downregulates IRF5 expression, reduces kynurenine generation, affects T cell function, inhibits TNBC progression and metastasis	*In vitro*(BT-549、MDA-MB-468)	(98)
Ginsenoside Rg3	0.8 ng/ml	/	TGF-β/SMAD	Downregulates TGF-β secretion, reduces CAF generation, promotes M1 macrophage enrichment, enhances immune infiltration	*In vitro* (4T1)	(100)
Peoniflorin	/	/	NF-κB/CCL2	Inhibits CCL2 secretion, reduces M2-type TAM infiltration and polarization, improves TIME	*In vivo*(MMTV-PyMT mouse )	(101)
Artemisinin	200 μg/100 μl/mice	14 days	NF-κB, PPARγ, chemokines (CCL5, CCL3)	Promotes macrophage polarization from M2 to M1, recruits CD8^+^ and CD4^+^ T cells, reshapes TIME	*In vitro* and *in vivo*(4T1、EMT-6、C127; BALB/c mice)	(103)
Paclitaxel	10 mg/kg	14 days	TLR4	Activates TLR4 pathway in TAMs, enhances antigen cross-presentation, promotes CD8^+^ T cell response	*In vivo* (C57BL/6、BALB/c mice)	(104)
Chlorogenic Acid	20 or 40 mg/kg	18 days	NF-κB, PD-L1	Inhibits NF-κB pathway, upregulates CD4^+^/CD8^+^ T cell ratio, reduces PD-L1 expression, delays tumor growth and metastasis	*In vivo* (BALB/c mice)	(108)
Baicalin	50 mg/kg	22 days	PI3Kγ/NF-κB	Promotes TAM polarization to M1 phenotype, increases secretion of TNF-α, KYNU and other effector factors, enhances M1 macrophage infiltration	*In vivo* (4T1 mice)	(106)
Curcumin	/	/	MAPK, STAT3, macrophage polarization	Activates MAPK pathway to promote DC maturation and T cell expansion; inhibits STAT3 expression, drives macrophage polarization from M2 to M1 phenotype	*In vitro* and *in vivo*	(110, 116)
Capsaicin	/	/	MAPK signaling cascade (MAPKKs, MEK1/2)	Modulates MAPK signaling, enhances immune surveillance of cancer cells, inhibits BC progression and drug resistance	*In vitro* and *in vivo*	(111)
Hesperidin	200mg/kg	14 days	JAK/STAT (STAT3, p-STAT3, JAK1, p-JAK1)	Downregulates key proteins in JAK/STAT pathway, promotes CD8^+^ T cell tumor infiltration, synergistically inhibits cancer cell proliferation, migration, and induces apoptosis	*In vitro* and *in vivo* (C57BL/6 mice; E0771)	(115)

### 5.1 Mechanistic actions of bioactive components in traditional Chinese medicine

Bioactive flavonoids, saponins, and terpenoids derived from TCM play pivotal roles in modulating the TIME of BC (92). These compounds exert their effects via multiple pathways, regulating immune cell functions while suppressing tumor growth and metastasis (93). For example, flavonoids such as quercetin, resveratrol, astragaloside IV, and isoliquiritigenin exhibit antioxidant, anti-angiogenic, and immunomodulatory properties, and are widely utilized in oncology (94). In experiments utilizing 4T1 cells and 4T1 murine models, quercetin was shown to exert antitumor effects by modulating the BC TIME through suppression of the IL-6/JAK2/STAT3 signaling pathway, resulting in reduced Treg cell populations (95). Resveratrol demonstrates multiple mechanisms against TNBC. Firstly, its anti-estrogenic activity directly inhibits the proliferation of ER-negative tumor cells. Secondly, *in vivo* studies revealed that resveratrol suppresses pulmonary metastasis by modulating the TIME—specifically by downregulating PD-1 expression on T cells in metastatic lung foci and promoting M1-type polarization of macrophages, thereby enhancing antitumor immunity and reducing immune evasion (96). In animal studies, co-delivery of hydroxymatrine and astragaloside IV via liposomes in combination with PD-1 inhibitors exhibited potent synergistic antitumor effects. The tumor inhibition rate (61.20%) and median survival (46 days) were 2.67 and 1.70 times higher, respectively, compared to the PD-1 monotherapy group (21.43%, 27 days). This synergistic mechanism was further validated *in vitro*, where the combination therapy significantly upregulated the expression of IFN-γ and granzyme B in CD8^+^ TILs, thereby systemically enhancing anti-BC immune responses (97). In studies targeting TNBC cell lines, isoliquiritigenin suppressed the IRF5/SLC7A5/IDO1 signaling axis, downregulating the expression of the key transcription factor IRF5 and significantly reducing the production of the tryptophan metabolite kynurenine. This modulation ultimately altered T cell immune functions, inhibiting TNBC progression and metastasis (98). Saponins, such as ginsenosides and paeoniflorin, modulate immune responses by enhancing macrophage phagocytic activity, promoting T cell proliferation and differentiation, and stimulating cytokine production (99). Ginsenoside Rg3 exhibits tumor-targeting activity against 4T1 BC cells and modulates the TIME by downregulating TGF-β secretion and altering the TGF-β/SMAD signaling axis, thereby reducing the recruitment of cancer-associated fibroblasts. This reprogramming favors the accumulation of M1-polarized macrophages and enhances immune cell infiltration into the tumor, effectively reversing the immunosuppressive TIME (100). Paeoniflorin, the primary active component of total paeony glycosides, exhibits potent anti-BC effects in the MMTV-PyMT murine model. Its mechanism involves the suppression of CCL2 secretion and inhibition of the NF-κB/CCL2 signaling axis, thereby reducing the infiltration and polarization of TAMs, especially the immunosuppressive M2 subtype. This leads to TIME improvement and inhibition of tumor growth and metastasis (101). Terpenoids such as artemisinin and paclitaxel demonstrate a broad range of effects, including antiproliferative, pro-autophagic, anti-angiogenic, and anti-metastatic activities (102). Artemisinin exerts dual regulation by activating the NF-κB pathway while inhibiting the PPARγ pathway, leading to the polarization of macrophages from an M2-like to an M1-like phenotype. The resulting M1 macrophages secrete chemokines such as CCL5 and CCL3, thereby enhancing the recruitment of various T cell subpopulations within the TIME. Specifically, this shift leads to an increase in CD8^+^ T cells from 2.8% to 11.5%, and CD4^+^ T cells from 55.2% to 66.0%, effectively reshaping the BC TIME (103). In TNBC mouse models, the antitumor mechanism of paclitaxel has been linked to its immunoregulatory effects on TAMs. By activating the TLR4 signaling pathway within TAMs, paclitaxel enhances their capacity for cross-presentation of antigens, significantly boosting the CD8^+^ T cell-mediated antitumor immune response (104). The divergent mechanisms of artemisinin and paclitaxel highlight the multifaceted immunomodulatory capacity of terpenoids, ranging from macrophage reprogramming to enhancement of antigen presentation.

### 5.2 Regulatory mechanisms of key signaling pathways

TCM modulates key immune-related signaling pathways in the TIME of BC, including NF-κB, MAPK, and JAK-STAT cascades (105). These pathways are essential for immune cell activation, polarization, and the enhancement of antitumor immunity (106). NF-κB is a pivotal transcription factor regulating immune and inflammatory responses (107). TCM-derived compounds exhibit cell-type-specific modulation of this pathway. In a 4T1 subcutaneous xenograft mouse model, chlorogenic acid significantly suppressed NF-κB signaling, increased the proportions of CD4^+^ and CD8^+^ T cells within the TIME, and downregulated PD-L1 expression. This reversal of the immunosuppressive TIME led to delayed tumor growth and metastasis (108). Wogonin modulates the PI3Kγ/NF-κB pathway to induce polarization of TAMs toward the M1 phenotype and enhances the secretion of M1-associated effectors such as TNF-α and KYNU. This immune reprogramming significantly increased M1 macrophage infiltration in the TIME and suppressed 4T1 breast tumor growth (106). These findings suggest that the same signaling pathway may elicit distinct immunomodulatory outcomes depending on the cellular context. The MAPK pathway plays a pivotal role in cell proliferation, differentiation, and survival (109). Herbal flavonoids such as curcumin activate MAPK signaling to promote DC maturation and T cell expansion, thereby enhancing antitumor immunity against BC (110). Capsaicin modulates the MAPK cascade and key downstream effectors including MAPKKs, MEK1, and MEK2, strengthening immune surveillance over tumor immune evasion. This mechanism contributes to its potent antitumor effects in BC, counteracting tumor progression, recurrence, and drug resistance (111). The JAK/STAT axis is central to cellular proliferation and immune regulation (112). Quercetin inhibits the activation of the JAK/STAT pathway, suppressing cancer cell survival, proliferation, and immune evasion. It restores immune surveillance, enhances T cell recognition and elimination of malignant cells, and promotes apoptosis of BC cells by upregulating p53 and pro-apoptotic protein BAX, while downregulating STAT3 and anti-apoptotic protein BCL2, thereby retarding tumor progression (113, 114). Naringin regulates the JAK/STAT cascade by downregulating key downstream targets such as STAT3, phosphorylated STAT3, JAK1, and phosphorylated JAK1, thereby enhancing antitumor immune responses. It increases CD8^+^ T cell infiltration into the tumor, synergistically inhibits cancer cell proliferation and migration, and promotes apoptosis, ultimately suppressing BC growth and metastasis (115). In murine studies, curcumin was shown to inhibit STAT3 expression, enhance intratumoral immune responses, and drive macrophage repolarization from M2 to M1 phenotype, thereby impairing BC cell growth and survival (116).

## 6 Preclinical and clinical evidence

To systematically verify the role of TCM in modulating the BC TIME, molecular mechanisms can first be elucidated through *in vitro* cell experiments, followed by *in vivo* functional validation in animal models, and clinical trials to provide preliminary translational evidence. This integrated evidence framework from basic research to clinical application provides scientific support for the clinical use of TCM.

### 6.1 *In vitro* cell-based studies

*In vitro* cell-based experiments provide a clear visualization of how TCM modulates the BC TIME and elucidate its mechanisms at the molecular and cellular levels. Gambogic acid (GA) has been reported to induce immunogenic cell death, releasing immune signaling molecules such as calreticulin and high-mobility group box protein 1 (HMGB1), thereby promoting the immune functions of DCs and CD8^+^ T cells, ultimately enhancing anti-breast cancer immunity (118). In immune cell modulation, polysaccharide monomers can stimulate the release of cytokines such as TNF-α and IL-6 *in vitro*, promoting the polarization of macrophages from the M2 to the M1 phenotype, which in turn inhibits tumor cell growth (119). Additionally, quercetin exhibits anti-BC effects, with two primary mechanisms: firstly, it induces γδ T cell differentiation into Vδ2 T cells to enhance immune regulation, thereby increasing cytotoxicity against BC cells; secondly, it activates the JAK/STAT1 signaling pathway, inhibiting PD-L1 expression and the binding of PD-L1 to PD-1 on T cell surfaces, thereby preventing T cell inhibition and restoring anti-tumor immunity (120). These *in vitro* experiments provide a preliminary molecular and cellular understanding of TCM’s anti-BC mechanisms, establishing a theoretical foundation for subsequent validation studies in animal models.

### 6.2 Validation studies in animal models

Animal studies investigating the modulation of the BC TIME by TCM have yielded significant results (121–123). These findings provide robust experimental support for the application of TCM in BC immunotherapy. BC mouse models, such as the 4T1 system, are indispensable tools for studying the TIME (124). The 4T1 cell line exhibits high invasiveness and metastatic potential, making it highly suitable for BC research (125). TCM herbs and their active ingredients, such as silymarin, platycodin D, and dihydrotanshinone I, have also demonstrated significant immunomodulatory effects in BC mouse models (126–128). For example, momordicine I inhibits M2 macrophage polarization via the MAPK and related pathways, remodeling the TIME and suppressing the growth of 4T1 tumors (129). Compound formulations also elucidate their anti-tumor mechanisms in BC mice through immunomodulation. For instance, Baoyuan Jiedu Decoction blocks the TGF-β/CCL-9 axis, reducing MDSC accumulation, improving the TIME, and inhibiting 4T1 metastasis (130). Overall, both single compounds and multi-herb formulations have demonstrated excellent immunomodulatory efficacy in BC mouse models, providing a solid foundation for clinical translation and boosting confidence in more effective immunotherapies.

### 6.3 Clinical translation

While numerous preclinical studies have elucidated the mechanisms by which TCM modulates the BC TIME, further human clinical trials are required for validation. A double-blind, randomized controlled trial involving 128 Chinese patients assessed the efficacy of Yanghe Decoction combined with neoadjuvant chemotherapy in patients with stage II-II BC. The trial design followed a single-center, randomized, placebo-controlled, double-blind approach. The results showed that the experimental group had a higher pathological complete response rate compared to the control group (55% vs. 30%) (131). Previous immunological studies demonstrated that the experimental group had significant increases in the infiltration of immune cells such as CD3^+^ and CD8^+^ T cells, as well as elevated levels of immune factors including IgG, IL-6, and IL-10, providing translational evidence for the use of Yanghe Decoction combined with neoadjuvant chemotherapy (131). Based on these findings, we can infer that Yanghe Decoction modulates the TIME and enhances the efficacy of neoadjuvant chemotherapy. However, there are limitations in this study, such as the inability of participants to undergo biopsy during neoadjuvant chemotherapy to clarify pathological subtypes, and the variability in the intake of Yanghe Decoction due to individual differences. Another multicenter cohort study evaluated the clinical efficacy and safety of combining San-Yin Decoction with conventional Western medicine in treating non-metastatic, recurrent TNBC (n=320/group). The study found that the recurrence and metastasis rates in the combination therapy group were lower than those in the Western medicine treatment group (12% vs. 33.33%), with immune function assessed every 3 months (NK, CD3, CD4, CD8, CD4/CD8) (132). This suggests that San-Yin Decoction significantly prolongs progression-free survival and overall survival in TNBC patients and has a positive effect on immune function. However, the study has limitations, including the lack of randomization and blinding. Although existing clinical trials demonstrate the potential of TCM in BC immunotherapy, especially when combined with chemotherapy or immunotherapy (e.g., immune checkpoint inhibitors), the clinical data available remains limited. Among the clinical trials registered with the China Clinical Trial Registry (ChiCTR), only one single-arm trial is directly related to the topic of this review. The trial, titled “Metabolomics Study of Compound Kushen Injection in Immunotherapy for Triple-Negative Breast Cancer” (Registration No.: ChiCTR1900026982), aims to systematically analyze the changes in metabolic profiles and pathways in patients before and after using Compound Kushen Injection. The goal is to identify key metabolites and pathways related to immune responses and perform targeted validation, providing empirical evidence for elucidating the potential metabolic mechanisms by which Compound Kushen Injection modulates the TIME of TNBC and enhances immunotherapy efficacy. Future efforts should focus on large-scale, randomized controlled trials to identify the optimal combination of TCM and modern immunotherapy strategies, thereby providing more effective treatment options for BC patients.

## 7 Potential of traditional Chinese medicine in breast cancer immunotherapy

In the field of BC immunotherapy, TCM has demonstrated potential as an adjunctive therapy to enhance immune responses, remodel the TIME, and improve overall therapeutic efficacy. Recent experimental studies have focused on combining TCM with immune checkpoint inhibitors (ICIs) or adoptive cell therapies. Notably, these combination therapies exhibit synergistic effects (“1 + 1>2”), providing strong impetus for further clinical exploration (133, 134). However, despite limited clinical data supporting the antitumor potential of combining TCM with immunotherapy, large-scale, randomized controlled trials are still lacking in BC immunotherapy, which limits the pace of its clinical translation (**Table 3**).

**Table 3:** Synergistic effects of traditional chinese medicine (TCM) with immunotherapies in breast cancer (BC).

Combination strategy	TCM/Active ingredient	Immunotherapy	Main synergistic mechanism & biological effect	Type of study	Reference
TCM + ICIs	Astragalus Polysaccharide	PD-1/PD-L1 Inhibitors	Downregulates PD-L1 expression on tumor cells, modulates microRNAs like miR-34a, enhances anti-tumor immune response.	*In vitro* experiments	(139, 140)
	Huagai Extract	PD-1/PD-L1 Antibody	Inhibits the conversion of CAFs to myoCAFs, significantly enhances T cell infiltration and activation in tumors, enhances immune attack against TNBC.	Animal experiments	(141)
	Paclitaxel	PD-1/PD-L1 Antibody	Regulates TAM polarization to M1 phenotype, reshapes immune microenvironment. Clinical trials show 27% improvement in overall survival and 13.6% increase in pathological complete response rate.	Clinical trials & mechanistic research	(104)
	Baicalin	Potential CTLA-4 Inhibitor	Upregulates CD8^+^ T cell and NK cell infiltration in the tumor microenvironment, increases CTLA-4 expression, suggesting enhanced sensitivity to CTLA-4 inhibitors.	Clinical observation & mechanistic analysis	(143)
	Traditional Chinese Medicine Formulation/Active Ingredient	Anti-CTLA-4 Treatment	Inhibits Tregs cell function, upregulates effector T cell proportion, enhances efficacy and may reduce immune-related adverse events.	Preclinical research	(87, 142)
TCM + Cell Immunotherapy	Gui Pi Decoction	NK cell therapy	Significantly enhances NK cell activity *in vitro* and tumor-killing function *in vivo*, with significant improvement observed in clinical cases.	Clinical case studies & *in vitro* research	(148)
	Various Phytochemicals	T cell/NK cell therapy	Promotes proliferation and activation of effector T cells and NK cells, inhibits immunosuppressive cells, enhances anti-tumor immunity.	*In vitro* & animal models	(149)
	Traditional Chinese Medicine Formula/Active Ingredient	T cell/NK cell therapy	Modulates cytokine secretion, reshapes TIME, enhances the efficacy of cell-based immunotherapy.	Mechanistic research	(89)

### 7.1 Combination of traditional Chinese medicine with immune checkpoint inhibitors

ICIs, such as antibodies targeting PD-1/PD-L1 or CTLA-4, work by releasing the immune “brakes” triggered by tumors, thereby restoring the antitumor functions of T cells (135). However, monotherapy with ICIs has shown only modest effects in BC, highlighting the need for strategies to enhance therapeutic efficacy (136). Increasing evidence suggests that TCM can enhance the efficacy of ICIs through multiple mechanisms (137, 138). For example, Bai Zhu polysaccharides enhance immune responses by downregulating PD-L1 and modulating microRNAs such as miR-34a (139, 140). Further animal studies show that Huagai extract, when combined with PD-1/PD-L1 antibodies, inhibits the transformation of CAFs into myofibroblasts (myoCAFs), significantly enhancing T cell infiltration and activation, and ultimately stimulating an immune response against TNBC (141). Recent clinical trials also confirm that the combination of paclitaxel with PD-1/PD-L1 antibodies significantly improves the prognosis of TNBC patients, with a 27% increase in overall survival and a 13.6% increase in pathological complete response rates. This efficacy is closely related to the immune modulatory function of paclitaxel in regulating the polarization of TAMs towards the M1 phenotype (104). In studies blocking CTLA-4, some research suggests that TCM formulations and their active ingredients can synergize with anti-CTLA-4 therapy by inhibiting Tregs and upregulating effector T cells, enhancing efficacy and reducing adverse effects (87, 142). Baicalin has been shown to increase the tumor infiltration levels of CD8^+^ T cells and NK cells in low-risk BC patients, while upregulating the expression of the immune checkpoint molecule CTLA-4. This suggests that baicalin may enhance the potential efficacy of CTLA-4 inhibitors in BC patients (143). In summary, these studies suggest that by modulating immune checkpoints, remodeling the tumor microenvironment, and enhancing immune responses, TCM provides promising avenues for combining with BC immunotherapy.

### 7.2 Synergistic effects of traditional Chinese medicine and cellular immunotherapy

Cellular immunotherapy, particularly T cell and NK cell therapies, has become a cornerstone of BC immunotherapy (144). T cell therapy selectively targets tumor cells by reprogramming autologous T cells (145), while NK cell therapy uses genetically engineered allogeneic NK cells to mediate tumor lysis (146). The efficacy of these cell-based therapies is limited by factors such as immune cell health, immune regulatory mediators, and intrinsic tumor resistance (147). Studies suggest that TCM can enhance the effectiveness of cellular immunotherapy through multiple mechanisms (148). Specific phytochemicals can promote the proliferation and activity of T cells and NK cells, while inhibiting immunosuppressive cells, thereby enhancing the antitumor effects of cellular immunotherapy (149). For instance, the combination of Gui Pi Decoction and NK cell therapy enhanced NK cell activity and achieved significant clinical improvement (148). Additionally, TCM can regulate cytokine secretion and remodel the BC TIME, further enhancing the effects of cellular immunotherapy (89). These findings provide mechanistic support for the combination of TCM and cellular immunotherapy, highlighting its therapeutic potential in BC treatment.

## 8 Application of omics and network pharmacology in traditional Chinese medicine research

In recent years, omics technologies and network pharmacology have emerged as pivotal research methods, each playing an increasingly important role in elucidating the complex mechanisms of TCM in treating breast cancer. When combined, these two approaches complement each other, contributing to the systematic understanding of how TCM regulates the BC TIME as a whole.

### 8.1 Multi-omics technologies

The integrated application of transcriptomics, proteomics, and metabolomics provides emerging research strategies to systematically elucidate how TCM modulates the TIME at the molecular level. Multi-omics technologies each have unique features in identifying how TCM affects the TIME in BC. Transcriptomics, by analyzing RNA expression changes, can reveal the regulatory effects of TCM components on the gene expression of immune-related cells. For example, matricaria ester has been found to significantly affect the transcriptome of BC-associated T cells, thereby regulating the TIME (150). Proteomics reveals how TCM regulates the TIME by altering interactions between immune cells and tumor cells (151). Metabolomics further reveals that certain TCM formulations can reshape the TIME by enhancing the immune surveillance function of key immune cells, such as CD8^+^ T cells, thereby reversing the tumor’s immune evasion state (152). These multi-omics evidences complement each other, enhancing the comprehensiveness of mechanistic research.

### 8.2 Network pharmacology

Network pharmacology is a powerful tool for analyzing the multi-target mechanisms of TCM formulas. By constructing a multi-dimensional network of “components-targets-pathways,” network pharmacology systematically reveals how TCM formulas regulate immune evasion in BC through multi-target and multi-pathway coordination. This method utilizes software screening and analysis to predict that several active components in TCM formulas may primarily target key molecules such as MAPK3, FOS, and ESR1, exerting anti-BC effects (153). For example, some TCM components can improve the body’s antitumor immune response by intervening in the PD-1/PD-L1 signaling axis (82). This strategy aids in the comprehensive understanding of the integrated regulatory mechanisms of TCM formulas at the multi-target and multi-pathway levels (154). This approach also reveals examples of novel mechanisms of action. For instance, the Fuzheng prescription has been shown to increase immune cell infiltration in the TIME, promote tumor cell apoptosis, and inhibit tumor growth (155). This method not only provides visual scientific evidence for the “multi-target” cancer treatment approach of TCM but also offers new directions for the design of subsequent experiments.

### 8.3 Integration of methodologies and future perspectives

The integration of omics and network pharmacology not only overcomes the limitations of single technologies but also further reveals the potential mechanisms by which TCM reverses tumor immunosuppressive states, such as through metabolic reprogramming (156). Multi-target action is a distinguishing feature of TCM treatment. TCM formulas often exert antitumor effects against BC by co-regulating multiple targets and pathways. The combination of omics and network pharmacology not only enhances the persuasiveness of experimental evidence but also clarifies the specific roles of various targets and pathways within the therapeutic network (157). Certain TCM formulations can simultaneously intervene in tumor immune evasion and cellular lipid metabolic reprogramming, demonstrating broad potential for application in BC immunotherapy (158). In the future, integrating more advanced techniques, such as single-cell sequencing and spatial transcriptomics, will enable the precise regulation of immune cell subpopulations and their interactions within the TIME at the single-cell and tissue levels. This will provide a solid theoretical foundation for TCM-based immunotherapy for BC (159, 160).

## 9 Challenges and prospects

TCM systematically regulates the complex TIME through its multi-target properties. This core concept is scientifically grounded and has demonstrated broad potential in BC immunotherapy (17). However, translating this potential into widespread clinical application still requires overcoming a series of challenges. We are confident that, in the near future, TCM will become a powerful adjunct to BC immunotherapy strategies.

### 9.1 Challenges currently faced

While TCM plays an important role in modulating the BC TIME, it still faces numerous challenges in current clinical applications. Firstly, due to tumor heterogeneity in BC, there is a lack of effective biomarkers for patient stratification, which imposes significant limitations on clinical treatment. The TIME and response to TCM vary among different molecular subtypes of BC. For example, TNBC responds differently to immune cell modulation compared to hormone receptor-positive BC, leading to varied effects of TCM across different subtypes (161, 162). Clinical trials on different molecular subtypes of BC, combined with multi-omics technologies, could be conducted to evaluate the corresponding clinical efficacy. Secondly, the standardization and quality control of TCM preparations remain major obstacles in TCM research. Due to differences in herb cultivation regions and preparation methods, the chemical characteristics of herbal extracts may vary significantly between batches, making it difficult to ensure the consistency and reproducibility of TCM efficacy (163). Closely related to this is the high environmental dependency of TCM’s immune regulatory effects. For example, astragalus polysaccharides show varying immune regulatory effects in different models, potentially promoting immune activation and antitumor effects under certain conditions, while inducing immune suppression or immune balance under other conditions (164). A full-chain quality control system from cultivation to production could be implemented, and modern technologies such as fingerprinting and biological potency assays should be used to evaluate the quality and efficacy of TCM preparations. Furthermore, the dose-response relationship of TCM is highly complex, as many TCM components exhibit non-linear effects. Low doses may be therapeutic, while high doses could lead to unintended and harmful effects, making it difficult to determine the optimal dosage (165). When combined with anticancer drugs, interactions between herbs and medications may occur, potentially diminishing the efficacy of the herbs or increasing the toxicity of the anticancer drugs, thus adding complexity and safety risks to treatment (166). Further research should be conducted to determine the effective dose range of drugs, establish safety evaluation systems for combination therapies, and enhance drug monitoring in clinical settings. Additionally, regulatory challenges for TCM are significant. The core issue lies in the mismatch between the multi-component, multi-target characteristics of TCM formulas and the current drug evaluation systems based on single components. To address this, integrating the unique experiences accumulated in TCM practice, such as innovations in operational procedures, scientific technology applications, and interdisciplinary collaboration, into the regulatory framework will strongly promote the transition of traditional medicines to a more standardized and scientific regulatory path (167). Lastly, many contemporary clinical studies face methodological limitations, including the lack of strict randomization, blinding, and control group designs. These studies often fail to eliminate potential biases, leading to concerns about the rigor and validity of the evidence (168). Future efforts should focus on improving study designs, controlling potential biases, and designing more rigorous, high-quality clinical trials, which will facilitate the clinical translation and application of TCM. In conclusion, while TCM has demonstrated potential in modulating the BC TIME, its clinical application still faces multiple challenges, including tumor heterogeneity, standardization, dose complexity, safety issues, regulatory barriers, and methodological limitations. There is an urgent need for more detailed molecular subtype mechanism studies, standardized herbal formulations, TCM regulatory framework development, and high-quality clinical trials to support its clinical application.

### 9.2 Immune modulatory potential of non-phytomedicinal traditional Chinese medicine therapies

In addition to herbal medicine, non-phytomedicinal TCM therapies such as acupuncture and moxibustion have also demonstrated potential value in BC rehabilitation and immune modulation. Existing studies suggest that their mechanisms may involve innate and adaptive immunity, by influencing the function and quantity of immune cells and mediators, thus improving tumor immune suppression and achieving immunotherapeutic effects (169). However, research in this field is still in its early stages. Most studies focus on alleviating cancer-related fatigue, improving quality of life, and reducing treatment side effects, with little investigation into direct immune modulation mechanisms (170–172). Furthermore, research on the regulation of the BC TIME by non-phytomedicinal therapies is still in its exploratory phase and faces methodological challenges, such as small sample sizes and imperfect control group designs, leading to a lack of reliability in the research conclusions (173). Future research could be further developed in the following ways: Firstly, it is recommended to design rigorous animal and cell experiments to systematically explore the specific mechanisms by which acupuncture and other non-phytomedicinal therapies regulate the BC TIME, such as identifying the key signaling pathways or molecular targets that affect specific immune cells, thereby reshaping the TIME (174). Secondly, clinical trial designs should adhere to methodological rigor, avoiding design flaws, and incorporate immune function-related indicators into the evaluation to enhance the credibility and scientific quality of the trials (175). Finally, due to the high heterogeneity of BC, combining cutting-edge technologies such as single-cell RNA sequencing and spatial transcriptomics will allow for the visualization of the dynamic effects of non-phytomedicinal therapies on the BC TIME, which will aid in guiding subsequent clinical treatments (176).

### 9.3 Future research directions

To thoroughly evaluate the efficacy and safety of traditional Chinese medicine in breast cancer immunotherapy, future multicenter, large-scale clinical trials are needed to further investigate the interactions of different drug combinations and their pharmacokinetic characteristics (177). These trials should include scientifically rigorous designs, objective diagnostic criteria, comparisons of different treatment regimens, and patient population selection (17). Conducting large-sample trials at multiple sites will increase accuracy and reliability, providing stronger evidence for the application of TCM in BC immunotherapy. For example, a Phase II randomized controlled trial could be designed for TNBC patients with specific TCM syndromes, comparing the efficacy and safety of [specific TCM formula] combined with anti-PD-1 to anti-PD-1 monotherapy. This trial will evaluate efficacy endpoints (such as pathological complete response rate, PFS, OS) and changes in specific components of the TIME to determine the advantages of combining TCM formulas with ICIs. Previous studies have shown that TCM can increase the sensitivity to immune inhibitors by inhibiting PD-1/PD-L1 expression, regulating immune cell functions, and improving the TIME. This combination with anti-PD-1 also demonstrates a synergistic effect (80). Additionally, a Phase III clinical trial for HER2-positive BC could be conducted to investigate the combined efficacy of [specific TCM formula] and standard treatment (such as trastuzumab). This trial will analyze the impact of combination therapy on tumor response rates, drug resistance, and quality of life, and evaluate the effect of TCM on regulating the TIME. Reports indicate that TCM combined with trastuzumab can improve quality of life for HER2-positive BC patients, reduce the cardiac toxicity of targeted drugs, enhance immune function, and potentially enhance the efficacy of targeted therapy by improving the tumor microenvironment (17, 178). Finally, to better integrate with modern immunotherapy, the combination of TCM with CAR-T cell therapy is also an important research direction for the future. A Phase III clinical trial for TNBC could explore how TCM enhances the efficacy of CAR-T cell therapy by modulating T cell and NK cell activity. Based on previous literature, we can hypothesize that TCM may assist CAR-T cell therapy by modulating the TIME (179). These specific clinical trial designs are expected to provide better clinical outcomes for BC immunotherapy and offer valuable insights for immunotherapy of other cancer types. Future research could expand into several new areas. Firstly, existing reviews have pointed out that gut microbiota plays a crucial role in enhancing the efficacy of ICI therapies (180). Building on this, we could explore the combined application of TCM and gut microbiota to verify whether modulating the gut microbiome can improve the TIME, providing additional support for BC immunotherapy. Moreover, the development of immunometabolism provides a new theoretical foundation for exploring how TCM regulates BC-related immune cell metabolism and reprograms the tumor microenvironment. Previous studies have indicated that the metabolic state of the tumor microenvironment affects immune cell function, which is ultimately closely linked to the success of immunotherapy (181). At the same time, integrating artificial intelligence with emerging disciplines such as microbiology and immunometabolism to conduct multi-center, large-scale clinical trials is expected to identify the most suitable TCM and immunotherapy combinations for patients, thereby improving the efficacy and efficiency of treatment. Ultimately, these strategies hold the potential to open up broader applications for cancer immunotherapy.

## 10 Conclusion

BC, the most common malignant tumor in women worldwide, has a TIME that plays a crucial role in the initiation, progression, and metastasis of the tumor. TCM modulates BC TIME through multiple targets and pathways. TCM, particularly Chinese herbal formulas, has been extensively demonstrated through experimental studies to reshape BC TIME, primarily by modulating the function and polarization of key immune cells, regulating immune checkpoints and cytokine networks, thereby inhibiting tumor immune escape and promoting anti-BC immune responses. Despite ample biological evidence revealing the potential of TCM in modulating BC TIME, its combined application with modern immunotherapy shows promising synergistic effects. The application of omics technologies and network pharmacology continues to elucidate its complex mechanisms of action. However, relevant clinical studies are still in the early stages, and there is a lack of large-scale, high-quality clinical trials to validate its efficacy and safety. The application of TCM in BC immunotherapy still faces multiple challenges, including tumor heterogeneity, dose complexity, and safety issues. Future work should focus on conducting multi-center, large-scale clinical studies to explore the integrative efficacy of TCM with modern immunotherapy, aiming to provide more cost-effective, low-toxicity treatment strategies for BC patients, improve response rates, and reduce adverse reactions, while also providing insights for other cancer types. In conclusion, TCM has demonstrated significant biological effects in BC immunotherapy. As clinical research progresses, it may become one of the key adjunctive strategies in BC immunotherapy.
